# Examination for the Factors Involving to Joint Effusion in Patients with Temporomandibular Disorders Using Magnetic Resonance Imaging

**DOI:** 10.3390/jimaging9050101

**Published:** 2023-05-16

**Authors:** Fumi Mizuhashi, Ichiro Ogura, Ryo Mizuhashi, Yuko Watarai, Makoto Oohashi, Tatsuhiro Suzuki, Hisato Saegusa

**Affiliations:** 1 Department of Removable Prosthodontics, The Nippon Dental University School of Life Dentistry at Niigata, Niigata 951-8580, Japan; 2 Department of Oral and Maxillofacial Radiology, The Nippon Dental University School of Life Dentistry at Niigata, Niigata 951-8580, Japan; 3 Comprehensive Dental Care, The Nippon Dental University Niigata Hospital, Niigata 951-1500, Japan; 4 Department of Dental Anesthesia and General Health Management, The Nippon Dental University School of Life Dentistry at Niigata, Niigata 951-8580, Japan; 5 Functional Occlusal Treatment, The Nippon Dental University Graduate School of Life Dentistry at Niigata, Niigata 951-8580, Japan

**Keywords:** temporomandibular disorders, magnetic resonance imaging, joint effusion

## Abstract

Background: This study investigated the factors involving joint effusion in patients with temporomandibular disorders. Methods: The magnetic resonance images of 131 temporomandibular joints (TMJs) of patients with temporomandibular disorders were evaluated. Gender, age, disease classification, duration of manifestation, muscle pain, TMJ pain, jaw opening disturbance, disc displacement with and without reduction, deformation of the articular disc, deformation of bone, and joint effusion were investigated. Differences in the appearance of symptoms and observations were evaluated using cross-tabulation. The differences in the amounts of synovial fluid in joint effusion vs. duration of manifestation were analyzed using the Kruskal–Wallis test. Multiple logistic regression analysis was performed to analyze the factors contributing to joint effusion. Results: Manifestation duration was significantly longer when joint effusion was not recognized (*p* < 0.05). Arthralgia and deformation of the articular disc were related to a high risk of joint effusion (*p* < 0.05). Conclusions: The results of this study suggest that joint effusion recognized in magnetic resonance imaging was easily observed when the manifestation duration was short, and arthralgia and deformation of the articular disc were related to a higher risk of joint effusion.

## 1. Introduction

The temporomandibular joint (TMJ) is a complex compound joint in the human body, and the joint cavity is divided into upper and lower compartments by the articular disc [1]. The articular disc shows a biconcave structure composed of dense fibrous connective tissue. The disc comprises a thicker anterior and posterior band and a thinner intermediate zone. The disc is attached to the temporal bone by the bilaminar zone, is highly vascularized, and innervated at the posterior part. The superior part of the lateral pterygoid muscle inserts onto the disc at the anterior part [2]. Disorders occurring in the TMJ are defined collectively as temporomandibular joint disorders (TMD). TMD is diagnosed based on the information obtained from the patient’s interview, standardized clinical examination, and TMJ imaging procedures. TMD is accompanied by major symptoms of pain in the muscle and TMJ as well as TMJ noises, such as clicking and crepitus, and a disturbance in jaw opening [3,4]. TMD is comprised different conditions with complex etiologies. Additionally, the symptoms of TMD also vary in intensity, and some symptoms improve spontaneously without any treatment while others persist, despite some treatments having been exhausted. TMD is classified by myofascial pain, arthralgia, disc displacement with reduction, disc displacement without reduction, osteoarthrosis, and so forth [5,6,7]. It is reported that 5–14% of the population experience clinical symptoms of TMD [8,9]. Another report mentioned that TMJ complaint is a common problematic distress that occurs in one-third of the population at some stage in their life [10]. A recent report in 2021 concluded that the prevalence of TMD was 31% for adults and 11% for children and adolescence [11]. TMD tends to occur between 20 and 40 years old [12]. Among the symptoms of TMD, disc dislocation on internal derangement is the most prevalent form [13]. Some reports state that disc displacement is found in 80–90% of symptomatic subjects [14,15]. Another report stated that muscle disorders were diagnosed in 56.9%, and disc displacements were recognized in 48.9% [16]. Thus, the frequency of TMD symptoms differs in the reported literature.

Magnetic resonance imaging (MRI) is a noninvasive imaging modality, considered the reference method for imaging soft tissue structures of the articular disc, synovial membrane, and lateral pterygoid muscle on the TMJ. Furthermore, MRI is the gold standard for diagnosing TMD [11,17,18]. MRI can also be used to assess hard tissues; however, the reliability is poor compared to CT or CBCT (cone beam computed tomography). MRI is adequate for observing disc displacement [19] and is often used for diagnosing TMD [20,21]. Diagnostic accuracy of MRI was reported as 98.5% for patients with anterior disc displacement without reduction, and that was reported as 61.6% for those with anterior disc displacement with a reduction [22]. Internal derangement is best evaluated with MRI by observing the articular disc and its location relative to the condyle in both closed mouth and mouth-opening positions. A displaced disc in MR images is a critical sign of TMJ internal derangement [23]. Disc displacement is diagnosed by proton density at high contrast, and the appearance of joint effusion is observed by a T2-emphasized image in MRI. In joint effusion, synovial fluid retention is observed as a hyperintense area in a T2-emphasized image and discussed to reflect the condition of TMJ inflammation [24]. Joint effusion can represent a local problem related to traumatic injuries and can be correlated with systemic diseases such as rheumatoid and psoriatic arthritis. In addition to these conditions, joint effusion is shown in relation to disc displacement and arthralgia [25]; however, effusion can even be observed in the TMJ without inflammation [26]. There is no consistent opinion concerning the interpretation of joint effusion. Instead, joint effusion is frequently encountered in diagnosing TMD with MRI. Additionally, the amount of synovial fluid in joint effusion varies among patients. This study aimed to clarify the meaning of joint effusion by examining the factors related to joint effusion in TMD patients.

## 2. Materials and Methods

This study reports on the TMJs of 131 TMD patients (18 male, 55 female, mean age: 45.5 ± 19.1 years) who came to the Nippon Dental University Niigata Hospital. The patients were diagnosed with TMD through medical examination by interview, clinical presentation, medical examination, and MRI findings. Table 1 shows the number of patients with myofascial pain, arthralgia, and a disturbance in mouth opening. Myofascial pain was confirmed by a complaint of pain in the masseter or temporalis muscles and the presence of pain by the palpation of the masseter or temporalis muscles. The number of patients with symptoms of myofascial pain was twice that of those with no symptoms. Arthralgia was confirmed by a complaint of TMJ pain and the presence of pain by palpations of the TMJ or mandibular movements. Twice as many patients had arthralgia symptoms as those with no symptoms of arthralgia. Mouth-opening disturbance was determined by the complaint of mouth-opening difficulty and a maximal mouth-opening measurement of less than 40 mm. The number of patients with a disturbance in mouth opening was small. Table 2 shows the duration of TMD manifestation. The duration of the manifestation was determined according to the medical examination by interview. One-third of patients came to the hospital within one month of the appearance of symptoms, and two-thirds came to the hospital within six months. This retrospective study was approved by the ethics committee of our institution. 

MRI (1.5 Tesla MR unit; EXCELART VantageMRT-2003; Canon Medical Systems, Otawara, Japan) with a surface coil for the TMJ included proton density-weighted sagittal and coronal imaging in the closed mouth position and the maximum mouth-opening position (repetition time/echo time 2000 ms/18 ms, field of view 130 mm × 130 mm, matrix size 256 × 224, and 1 acquisition). T2-weighted sagittal and coronal imaging in the closed mouth position and the maximum mouth-opening position (repetition time/echo time 3500 ms/100 ms, field of view 130 mm × 130 mm, matrix size 256 × 192, and 2 acquisitions) was also included [27,28]. Joint effusion, disc displacement, disc deformation, and bone deformation were analyzed by MR imaging. Joint effusion was recognized as the hyperintense area of superior or inferior articular cavities on the T2-emphasized image. In this study, the amount of synovial fluid in joint effusion was evaluated by classifying its presentation into four degrees; Grade 0 (no fluid), Grade 1 (fluid with punctiform or filamentous), Glade 2 (fluid with cingulate), and Glade 3 (fluid with plenitude). Disc displacement was recognized on the MR image and the anterior or posterior disc displacement was diagnosed on sagittal oblique cross-section imaging. Inside or outside disc displacement was determined by coronal cross-section imaging of proton density-weighted images. The deformation of the disc and bone was confirmed by sagittal oblique cross-section imaging and coronal cross-section imaging of proton density-weighted images. Two radiologists independently evaluated all MR images, and any differences were resolved by forced consensus. Nonagreements requiring forced consensus were 2.29% (3/131) in disc displacement, 0% (0/131) in disc displacement with or without reduction, 4.58% (6/131) in disc deformation, 3.82% (5/131) in bone deformation, and 1.53% (2/131) in joint effusion.

This study examined the proportion of symptoms and observations of the TMJs, and the factors that could be considered to contribute to the appearance of joint effusion (gender, age, myofascial pain, arthralgia, disturbance of mouth opening, disc displacement, disc deformation, and bone deformation) were compared. The proportion of myofascial pain, arthralgia, disturbance in mouth opening, disc displacement, disc deformation, bone deformation, and joint effusion was analyzed by the existence of symptoms and observations using cross-tabulation. The difference in the amount of synovial fluid in joint effusion (the amount of synovial fluid was classified from Grade 0 to Glade 3) by the duration of manifestation was analyzed using the Kruskal–Wallis test. Additionally, multiple logistic regression analyses were performed to analyze the factors contributing to joint effusion: gender, age, myofascial pain, arthralgia, disturbance in mouth opening, disc displacement, disc deformation, and bone deformation. For the multiple logistic regression analysis, the symptoms and observations were categorized as follows: gender (man: 0, woman: 1), myofascial pain (nonexistence of symptom: 0, the existence of symptom: 1), arthralgia (nonexistence of symptom: 0, the existence of symptom: 1), disturbance in mouth opening (nonexistence of symptom: 0, the existence of symptom: 1), disc displacement (normal disc position: 0, disc displacement: 1), disc deformation (normal disc: 0, deformed disc: 1), and bone deformation (normal bone: 0, deformed bone: 1). Statistical analysis was performed using statistical analysis software (SPSS 17.0, SPSS JAPAN, Tokyo, Japan), and differences of α < 0.05 were considered significant.

## 3. Results

**Figure 1:**
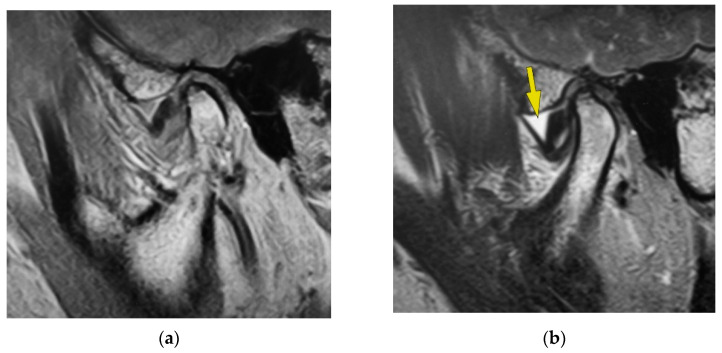

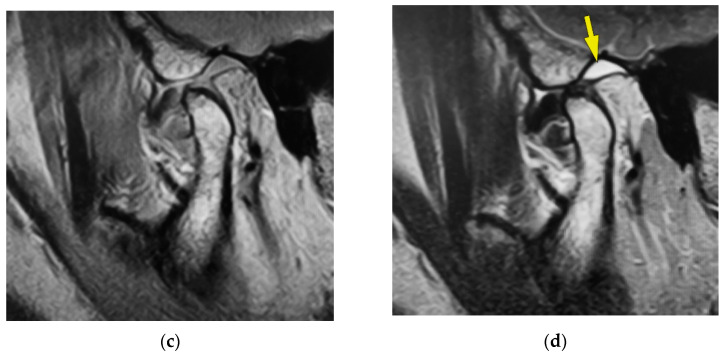
MR image of one patient with disc displacement without reduction. (**a**) Proton density-weighted sagittal oblique cross-section imaging at the closed mouth position. (**b**) T2-weighted sagittal oblique cross-section imaging at the closed mouth position. The yellow arrow indicates the area of joint effusion. (**c**) Proton density-weighted sagittal oblique cross-section imaging in the maximum mouth-opening position. (**d**) T2-weighted sagittal oblique cross-section imaging in the maximum mouth-opening position. The yellow arrow indicates the area of joint effusion.

Figure 1 indicates the MR image of one patient in this study with recognized disc displacement without reduction. This MR image is of the left temporomandibular joint of a 48-year-old woman with left temporomandibular joint pain. Proton density-weighted sagittal oblique cross-section imaging in the closed mouth position shows disc displacement (Figure 1a). The articular disc is recognized at the anterior position of the mandibular condyle, and disc deformation was also observed. Bone deformation was not recognized in this image. The T2-weighted sagittal oblique cross-section imaging in the closed mouth position shows temporomandibular joint effusion, as shown by the yellow arrow (Figure 1b). The part observed as the hyperintense area is joint effusion, observed at the superior articular cavity. The amount of synovial fluid in the joint effusion was observed as fluid with plenitude. Proton density-weighted sagittal oblique cross-section imaging at the maximum mouth-opening position shows the articular disc at the anterior position of the mandibular condyle, namely, disc displacement without reduction (Figure 1c). T2-weighted sagittal oblique cross-section imaging at the maximum mouth-opening position shows temporomandibular joint effusion, as shown by the yellow arrow (Figure 1d). The part of the hyperintense area is joint effusion, as observed at the superior articular cavity. The amount of synovial fluid on joint effusion was recognized as fluid with plenitude.

The results of the multiple logistic regression analysis are shown in Table 3. Some factors that were considered to contribute to the appearance of joint effusion were gender, age, myofascial pain, arthralgia, mouth-opening disturbance, disc displacement, disc deformation, and bone deformation. Among these factors, arthralgia was related to a higher risk of joint effusion, and the odds ratio was 2.602 (95% confidence interval: 1.122–6.033, *p* < 0.05). Additionally, disc deformation was also related to a higher risk of joint effusion, and the odds ratio was 3.371 (95% confidence interval: 1.278–8.893, *p* < 0.05). The odds ratio of gender was 4.000 (95% confidence interval: 1.270–12.597, *p* < 0.05). Age, myofascial pain, mouth-opening disturbance, disc displacement, and bone deformation were not recognized as the related factors to a higher risk of joint effusion.

**Figure 2:**
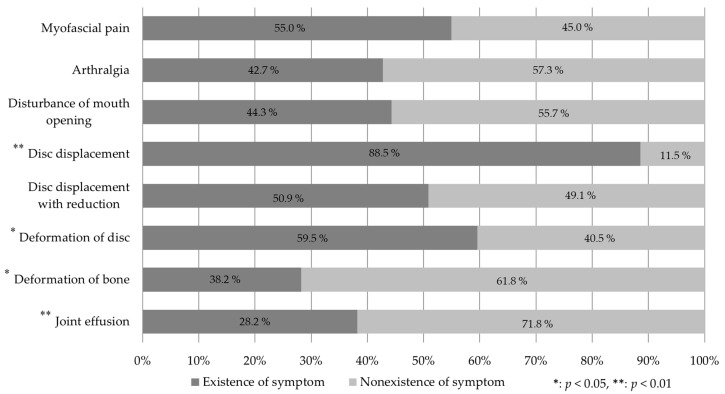
Proportion of symptom and observations.

The symptom proportion results are shown in Figure 2. In the cross-tabulation, the proportion of disc displacement (88.5%) was significantly larger than that of normal disc placement (11.5%) (χ^2^(1) = 77.87, *p* < 0.01), and the deformation of the disc (59.5%) was significantly more frequent than normal disc formation (40.5%) (χ^2^(1) = 4.77, *p* < 0.05). Bone deformation (38.2%) was significantly less frequent than normal bone formation (61.8%) (χ^2^(1) = 24.80, *p* < 0.01), and the proportion of joint effusion (28.2%) was significantly smaller than that without joint effusion (71.8%) (χ^2^(1) = 7.34, *p* < 0.01). The other symptoms and observations, such as myofascial pain, arthralgia, mouth-opening disturbance, and disc displacement without reduction, were found in about half of the TMJs in this study. 

**Figure 3:**
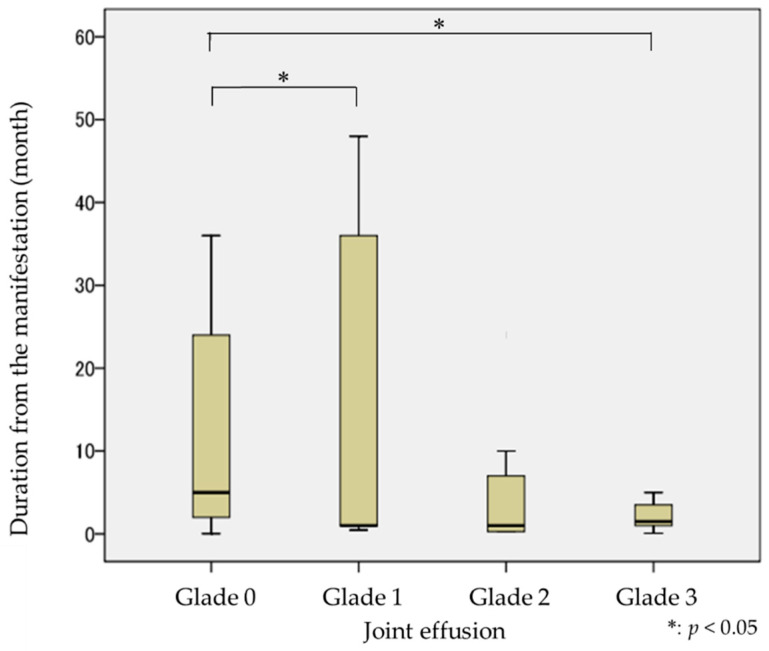
Amount of synovial fluid on joint effusion by the duration from the manifestation.

The difference in synovial fluid in joint effusion, according to the duration of manifestation, is shown in Figure 3. The median value for manifestation duration in Grade 0 (no fluid) was four months; in Grade 1 (fluid with punctiform or filamentous), it was one month; in Grade 2 (fluid with cingulate), it was three months; and in Grade 3 (fluid with plenitude), it was two months. There were statistically significant differences between Grade 0 and Grade 1 (*p* < 0.05), and Grade 0 and Grade 3 (*p* < 0.05), concerning the duration of manifestation. The amount of synovial fluid in joint effusion observed in MR images was greater for shorter manifestation durations.

## 4. Discussion

Joint effusion is observed as the retention of synovial fluid, as shown on T2-emphasized images from MRI, and reflects the condition of the inflammation of the TMJ [24]. On the other hand, joint effusion could be observed even in TMJs without inflammation [26]. Joint effusion is frequently observed in the MR images of TMD patients in clinical situations; however, the interpretation of joint effusion is not clear enough. This study examined the factors relating to joint effusion in TMD patients according to interview, clinical presentation, medical examination, and MRI findings. 

MRI is adequate for observing disc displacement [19] and for the diagnosis of TMD [20,21] because it references the soft tissue structures of the articular disc, synovial membrane, and lateral pterygoid muscle in the TMJ [11,17,18]. MRI with a surface coil for the TMJ included proton density-weighted sagittal and coronal imaging in the closed mouth position and the maximum mouth-opening position. In MRI, we used a surface coil for the TMJ, not the head coil, because of its high sensitivity. Dynamic MRI has been developed recently in addition to static MRI [29,30,31]. Dynamic MRI can provide additional information concerning disc and condyle mobility, disc reduction, and topographic changes in the disc–condyle relationship during mouth movement [32,33]. Dynamic MRI added superior information regarding the movement patterns of the condyle and disc in different types of TMJ internal derangement [34]. However, a dynamic sequence was not performed in this study because our MR unit is a 1.5-Tesla, not a 3-Tesla.

In this study, there were three times more female patients than males, and this tendency is the same as in previous reports [35]. It is reported that the prevalence of TMD is higher in females due to their increased duration of TMD symptoms; at any given moment, more females have TMJ symptoms [36]. Therefore, there were more females than males in this study. The proportions of disc displacement and deformation were larger than that of normal disc placement or formation, and this tendency is similar to other reports [15,37]. Disc displacement was found in 88.5% of the TMJs investigated in this study. This percentage of the prevalence of disc displacement was almost the same as the previous reports that reported the prevalence of disc displacement to be 80–90% of symptomatic subjects [14,15]. In this study, myofascial pain, arthralgia, and mouth-opening disturbance were found in about half of the TMJs. The proportion of patients reporting myofascial pain in this study was similar to another report that reported muscle disorders in 56.9% [16]. The subjects with bone deformation or the appearance of joint effusion on the TMJs were not large in this study.

The amount of synovial fluid in joint effusion was investigated as the hyperintense area of the superior or inferior articular cavities in T2-emphasized MR images, and the amount of synovial fluid in joint effusion was classified from no fluid (Grade 0) to fluid with plenitude (Grade 3). The differences in the grade of joint effusion vs. manifestation duration were analyzed to clarify the timing of joint effusion appearance. The results indicated that the duration of manifestation was longest in TMJs with no fluid on the superior or inferior articular cavities compared to that with fluid with punctiform or filamentous and fluid with plenitude on the superior or inferior articular cavities. Namely, the amount of synovial fluid in joint effusion differed with manifestation duration. The amount of synovial fluid in joint effusion was larger when the manifestation duration was short. This result suggested that joint effusion reflects the inflammation of the articular cavities in the early stages.

The factors contributing to joint effusion were gender, age, myofascial pain, arthralgia, mouth-opening disturbance, disc displacement, disc deformation, and bone deformation. These factors were investigated using multiple logistic regression analysis, and the result made it clear that the arthralgia and deformation of the disc were related to a higher risk of the appearance of joint effusion. Although the odds ratio for gender was also significantly high, this result was caused by the fact that there were three times more females than males. This result supports the previous studies that demonstrated the relationship between joint effusion and arthralgia [24,38] and is consistent with the reports indicating no relationship between joint effusion and arthralgia [25,39]. Joint effusion has been suggested as a surrogate for an inflammatory process because joint effusion might activate or sensitize nociceptive afferent neurons within the joint, resulting in increased articular pressure that causes mechanical trauma [40,41,42,43]. Therefore, joint effusion would be caused by the condition associated with TMJ pain. The result of this study suggested that joint effusion would not occur when myofascial pain is indicated but by the change in TMJ condition, such as arthralgia and disc deformation. Bone deformation did not contribute to joint effusion; therefore, it was suggested that joint effusion occurs early in the change in TMJ condition. The exudate in articular cavities decreased as acute inflammation improved. Further study is needed to investigate changes in the amount of synovial fluid in joint effusion according to the improvement of TMJ symptoms.

Joint effusion is frequently encountered in the diagnosis of TMD in MR images. However, joint effusion is not well understood. This study investigated the factors that influence the appearance of joint effusion in TMD patients. The results suggested that a greater presence of synovial fluid in joint effusion occurs in the early stages of acute inflammation of the TMJ. Joint effusion could be recognized with the conditions of arthralgia or deformation of the disc. It was clear that joint effusion in MRI could present as one of the parameters for the assessment of the TMD condition in patients. However, further research is required to clarify the meaning of joint effusion. The limitations of this study were that the TMJs of the TMD patients were observed only before the treatment. If the changes in synovial fluid in joint effusion were evaluated in MRI with the change in symptoms and observations, the meaning of joint effusion could be more precise. In future research, the change in the joint effusion of each subject after treatment should be investigated. Additionally, examination using dynamic MRI would be effective for improving knowledge.

## 5. Conclusions

This study investigated the factors relating to the joint effusion of TMD patients using MRI. As a result, joint effusion tended to be observed in the TMJ, and the duration of manifestation was short. It is suggested that arthralgia and deformation of the disc are related to a higher risk of joint effusion manifestation.

## Figures and Tables

**Table 1:** Number of patients with different symptoms.

Symptom	Number
Myofascial pain	
Existence of symptom	53
Nonexistence of symptom	20
Arthralgia	
Existence of symptom	51
Nonexistence of symptom	22
Disturbance of mouth opening	
Existence of symptom	32
Nonexistence of symptom	41

**Table 2:** Duration from the manifestation.

Duration from the Manifestation	Number
0–1 month	27
2–3 month	14
4–6 month	11
7–12 month	8
24–36 month	9
48–60 month	4

**Table 3:** Result of multiplex logistic-regression analysis.

Factor	Odds Ratio	95% Confidence Interval	*p* Value
Gender	4.000	1.270–12.597	0.018 *
Age	0.981	0.959–1.004	0.098
Myofascial pain	0.446	0.180–1.106	0.082
Arthralgia	2.602	1.122–6.033	0.026 *
Disturbance of mouth opening	0.882	0.392–1.987	0.762
Disc displacement	0.899	0.142–5.694	0.910
Deformation of disc	3.371	1.278–8.893	0.014 *
Deformation of bone	1.523	0.633–3.663	0.348

* *p* < 0.05.

## Data Availability

The data presented in this study are available on request from the corresponding author.
